# The Metabolic Requirements of Th2 Cell Differentiation

**DOI:** 10.3389/fimmu.2019.02318

**Published:** 2019-09-27

**Authors:** Julian M. Stark, Christopher A. Tibbitt, Jonathan M. Coquet

**Affiliations:** Department of Microbiology, Tumor and Cell Biology, Karolinska Institutet, Stockholm, Sweden

**Keywords:** Th2, PPAR-γ, lipid metabolism, glycolysis, mTOR

## Abstract

Upon activation, naïve CD4^+^ T cells differentiate into a number of specialized T helper (Th) cell subsets. Th2 cells are central players in immunity to helminths and are implicated in mediating the inflammatory pathology associated with allergies. The differentiation of Th2 cells is dependent on transcription factors such as GATA3 and STAT6, which prime Th2 cells for the secretion of interleukin- (IL-) 4, IL-5, and IL-13. Several lines of work now suggest that differentiating Th2 cells in the lymph node are potent IL-4 cytokine producers, but do not become competent IL-5- and IL-13-producing cells until after receiving cues from non-lymphoid tissue. It is evident that Th2 cells that enter tissues undergo considerable changes in chromatin architecture and gene expression, and that over this time, the metabolic requirements of these cells change considerably. Herein, we discuss the metabolic requirements of Th2 cells during their early and late differentiation, focusing on the impact of glucose and lipid metabolism, mTOR activation, the nuclear receptor PPAR-γ and several metabolites.

## Introduction

CD4 T cells are central mediators of immunity to infections and cancers. Pioneering studies by Mosmann and Coffman identified mouse CD4 T cell clones with distinct functional properties that they termed T helper (Th) 1 and Th2 cells (1). Over 30 years of research has since defined several additional subsets of CD4 T cells including Th17, Tfh, and T regulatory (Treg) cells. Th2 cells are defined by the expression of lineage-defining transcription factors including GATA3 and STAT6, surface molecules such as IL-33R and CCR8 and the effector cytokines IL-4, IL-5, and IL-13 (2). Through the secretion of IL-4, IL-5, and IL-13, Th2 cells promote B cell isotype class switching to IgG1 and IgE (3), induce the alternative activation (M2) phenotype in macrophages (4, 5), induce eosinophil recruitment and promote mucus secretion through the process of goblet cell metaplasia (6, 7). These effector functions have been shown to support immunity to helminths, venoms, certain bacterial infections, and are also beneficial in tissue healing (8, 9). However, Th2 cell-mediated immune responses are also implicated in allergic disorders including asthma, atopic dermatitis, chronic rhinitis, and some forms of gut disorders including ulcerative colitis (10–12). The rise in Th2 cell-mediated disorders has become especially apparent in the past 50 years and represents a significant and growing health and economic challenge.

## The Process of Th2 Cell Differentiation

Th2 cell differentiation from naïve CD4 T cells is typically dependent on the presence of interleukin-4 (IL-4) in the local cytokine milieu. Ligation of the IL-4R induces JAK1/3 mediated phosphorylation and dimerization of Signal Transducer and Activator of Transcription-6 (STAT6) (13). pSTAT6 dimers then translocate to the nucleus and induce expression of GATA3; the so-called “master” regulator of the Th2 cell lineage. GATA3 is sufficient to induce the Th2 cell phenotype, since for instance, enforced retroviral expression of GATA3 results in IL-4 production in Th1 cells (14, 15). Furthermore, GATA3-deficient T helper cells have impaired Th2 cell differentiation as shown in *in vitro* and *in vivo* studies (16–19). Expression of GATA3 results in profound modifications to the chromatin landscape across the *Il4/Il5/Rad50/Il13* locus at a number of well-characterized sites including several enhancer sites and a locus control region located in *Rad50* (20–23). Together with STAT6, this creates an “active” chromatin hub that allows co-ordinated expression of Th2 cell effector cytokines and a positive feedback loop through which GATA3-induced IL-4 maintains Th2 cell identity (24–26). Other genes important in the later stages of Th2 cell differentiation are also bound by GATA3 including the *Il1rl1* gene encoding a subunit of the IL-33R known as ST2, and the chemokine receptor *Ccr8* (27, 28).

Although the canonical pathway of Th2 cell differentiation is thought to proceed through GATA3 and STAT6, a number of non-classical pathways are also thought to be important during the early stages of Th2 cell differentiation, exemplified by the presence of IL-4^+^ and IL-13^+^ Th2 cells in STAT6-deficient mice (29). IL-2, induced upon TCR activation, has been shown to be capable of driving IL-4 production in T helper cells in an IL-4R-independent manner (30, 31). Triggering of the IL-2R results in activation of STAT5, with STAT5A being the most dominant isoform inducing downstream IL-4 expression (16). Support for the role of STAT5A in Th2 cell differentiation comes from studies of double STAT5A/STAT6-deficient mice that have further impairments in Th2 cell responses when compared to single STAT6-deficient mice (31, 32). STAT3 was also shown to be important for Th2 cell differentiation by guiding STAT6 to critical Th2 cell gene loci (33). Other studies have shown roles for a number of transcription factors in type 2 cytokine production including c-Maf, NF-κB, and IRF4 during the early stages of Th2 cell differentiation (34–36). Therefore, Th2 cell fate is determined by a complex network of transcription factors that together shape and promote naïve cells to adopt and maintain the Th2 cell phenotype.

## Timed Cytokine Expression in Th2 Cells

The Th2 cell effector cytokine genes *Il4, Il13*, and *Il5* are positioned together with *Rad50* (Chromosome 5 in humans; Chromosome 11 in mice), which contains a locus control region that co-ordinates at least *Il4* and *Il13* expression (24, 37). Despite the close proximity of these genes, their expression is exquisitely timed and not always concomitant. IL-4 expression is clearly detected in activated CD4 T cells in the lymph node, although several studies have shown that these cytokine-secreting cells are a mixed population of Th2 cells and Tfh cells, which require only low levels of GATA3 expression together with c-Maf (38–40). Meanwhile, IL-5 and IL-13 expression is a feature of Th2 cells only once these cells enter inflamed tissues such as the lungs (39, 41, 42). In response to house dust mite (HDM) allergens, airway Th2 cells tended to express less *Il4* mRNA than their lymph node counterparts, suggesting that IL-4 is the dominant cytokine in the lymph node, while IL-5 and IL-13 are the dominant Th2 cell-derived cytokines in tissues. This distinct timing means that the absence of IL-4 or IL-13 has distinct functional consequences (37, 39, 41, 42). For instance, IL-4-deficient mice were found to clear the helminth *Nippostrongylus brasiliensis* more rapidly despite reduced IgE titers (43). In contrast, IL-13-deficient mice had significantly higher worm counts and took longer to clear infections despite no defect in IgE production. Similar responses were observed in models of *Trichuris muris* and *Heligmosomoides polygyrus* infection (44–46). Hence, the quality of Th2 cells changes over time and their function depends on the tissue context.

## Metabolic Pathways Important to T helper Cells

Generation of energy and biosynthesis of metabolites is critical to the activation, proliferation and differentiation of T helper cells (47). Naïve CD4 T cells favor the generation of energy via mitochondrial pathways (48). The tricarboxylic acid (TCA) cycle is a highly efficient means of converting acetyl-CoA into carbon dioxide and ATP and leads to the generation of NADH and FADH_2_ in the inner membrane of the mitochondria (47). These two products are vital for the transfer of electrons in the electron transfer chain (ETC) via complexes I–IV. Given its greater efficiency in terms of ATP generation compared to glycolysis, the TCA cycle is able to meet the energy needs of long lived cells such as naïve CD4 T cells (49, 50).

Fatty acid oxidation is a means by which T helper cells can convert fatty acids for the generation of significant amounts of energy. The initial steps occur in the cytosol using ATP to generate fatty acid acyl-CoA, which is transported into the mitochondria via carnitine palmitoyltransferase I (CPT-1). Beta oxidation of fatty acids then produces acetyl-CoA, NADH and FADH_2_ (47), which all help to fuel the TCA cycle.

During initial activation, glycolysis becomes the dominant metabolic pathway in T helper cells (51). Under the control of transcription factors such as c-Myc and HIF-1α, extracellular glucose is taken up and catabolized to pyruvate, which yields 2 ATP per molecule of glucose (52–54) and provides a source of acetyl-CoA for the TCA cycle. Glycolysis also rapidly provides NADH and a range of intermediates, which are useful in anabolic pathways including nucleotide, amino acid and fatty acid biosynthesis (47). Reduction of pyruvate to lactate is also important to replenish NAD^+^ levels within the cell.

In addition to oxidation of lipids, *de novo* fatty acid synthesis needs to take place and is controlled by enzymes including sterol regulatory element-binding protein (SREBP), fatty acid synthase (FAS) and Acetyl-CoA carboxylase (ACC) (55). Straight chain and branched fatty acids are produced from products generated during glycolysis, the TCA cycle and the pentose phosphate pathway (47). For straight chain fatty acids, citrate is exported from the mitochondria and converted into acetyl-CoA in the cytosol. Following carboxylation by ACC, it can be further extended by FAS in a NADPH-dependent mechanism to varying chain lengths. In order to produce branched forms, amino acids such as leucine or valine are required while fatty acids may also be combined with glycerol to form triacylglycerides and phospholipids (56).

All metabolic pathways are highly intertwined since products and intermediates from one pathway can function as key synthetic precursors in other pathways. These pathways not only promote cell division, survival and expansion, but metabolites and co-factors can also directly influence gene expression by modifying chromatin, acting as ligands for transcription factors and influencing the stability of cytokine mRNAs in the cytosol.

Below, we review the literature on the metabolic demands of CD4 T cells, in particular as they relate to Th2 cells. We address what is known of early Th2 cell differentiation, which primarily occurs in the context of the lymph node and then address metabolic adaptations of Th2 cells in the context of tissue immunity.

## Metabolic Changes During Early Activation of Th2 Cells

### Promotion of Th2 Cell Differentiation by Mammalian Target of Rapamycin (mTOR)

**Figure 1:**
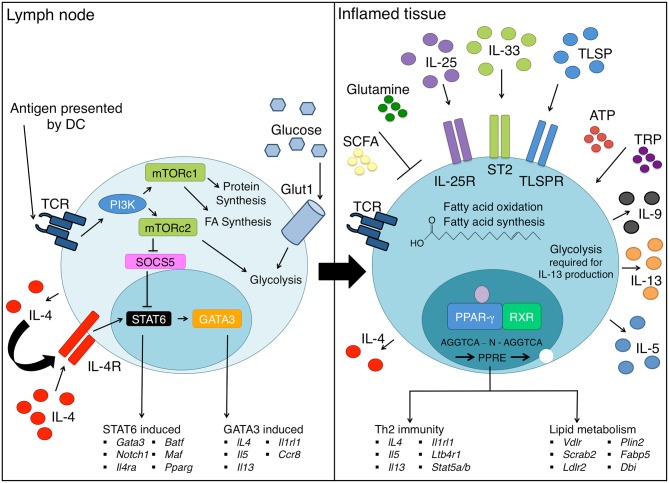
Th2 cell differentiation requires extensive metabolic reprogramming. Upon encountering cognate antigen in the lymph node, naive CD4 T helper cells differentiate into Th2 cells under the influence of the IL-4-STAT6-GATA3 axis. Concurrently, changes in energy requirements occur as a Th2 cell differentiates, which requires an increase in glucose uptake via GLUT1 and marked upregulation of glycolysis. mTORc1 senses nutrient availability and co-ordinates metabolism in T helper cells, and mTORc2 promotes Th2 cell differentiation through several mechanisms. As Th2 cells enter inflamed tissue sites such as the lung, they continue to differentiate through exposure to a range of inflammatory cytokines including TSLP, IL-25, and IL-33 that promote production of effector cytokines such as IL-5, IL-9, and IL-13. In addition, PPAR-γ drives expression of lipid metabolic genes such as *Fabp4* and *Vdlr* as well as genes critical to Th2 cell effector functions, such as ST2 (*Il1rl1*) and *Il5*. Extracellular metabolites present in the tissues including SCFA, ATP, and TRP can further promote Th2 cell differentiation and/or function. On the other hand, glutamine obtained from the diet potentiates Th1 cells at the expense of Th2 cells.

In early and probably also later differentiation of T helper cells, coordination of cell growth, proliferation and metabolism is mediated by the kinase mTOR (57) (Figure 1). The mTOR complex monitors nutrient availability and integrates signals from growth factors and cytokine receptors to regulate glucose, amino acid and lipid metabolism. mTOR complex1 (mTORc1) is formed with the scaffolding protein regulatory associated protein of mTOR (RAPTOR) while mTOR complex 2 (mTORc2) uses Rapamycin-insensitive companion of mammalian target of rapamycin (RICTOR) as a scaffold (58). Differentiation toward the effector Th cell lineages Th1, Th2, and Th17 is known to be reliant on mTOR activity, while inhibition of mTOR with rapamycin has been shown to favor Treg cell differentiation (59–61). All effector lineages including Th2 cells have been shown to require mTORc1 activation, since deletion of RAPTOR and thereby mTORc1 potently inhibits effector differentiation (62).

A number of studies have also highlighted a specific role for mTORc2 in Th2 cell differentiation. mTORc2 inhibits suppressor of cytokine signaling-5 (SOCS5) (63), which in turn suppresses IL-4-dependent STAT6 signaling to block Th2 cell differentiation (64). SGK1, a downstream target of mTORc2, promotes Th2 cell lineage commitment while blocking Th1 cell development (65). Furthermore, deletion of the GTPase RhoA, another mTORc2 target leads to decreased glycolysis and IL-4 production (66). Thus, while there is a clear requirement for mTORc1 in early Th2 cell development, signals downstream of mTORc2 seems to have distinct positive effects on the differentiation of Th2 cells.

### Early Induction of Glycolysis in T helper Cells

Naïve T cells rely primarily on oxidative lipid metabolism as they recirculate among lymph nodes (51, 52). However, naïve T cells are poised for a rapid switch to effector cell metabolism by accumulating untranslated mRNAs required for glycolysis and fatty acid synthesis (67). Activation of T cells through the T cell receptor, co-stimulatory ligands and cytokine receptors is followed by expansion, differentiation and production of effector cytokines; processes which place a great metabolic pressure on cells.

Cells can upregulate glycolysis at faster rates than oxidative phosphorylation, as glycolysis requires no mitochondrial growth (47). The high rate of glycolysis in effector T cells requires activation of mTOR, HIF-1α and increased expression of glucose transporters like Glut1, which is essential for CD4 but not for CD8 T cells (68, 69). Glut1 is translocated to the cell surface upon activation, a process mediated through the phosphatidylinositol 3-kinase (PI3K)-AKT pathway (70) and Myc expression (53). Glycolysis supports T cell activation in many ways; for instance by supporting epigenetic modifications through lactate dehydrogenase A (54) and by supplying dividing cells with many side products required for division and growth. Effector T helper cell subsets including Th1, Th2, and Th17 are all highly dependent on glycolysis for growth and function, and a small subset of Treg cells has also been shown to be highly glycolytic (61). Effector T helper cells have been shown to undergo various levels of glycolysis in *in vitro* assays and Th2 cells express the most Glut1 and appear the most glycolytic, when analyzed via Seahorse Analyzer (41, 51), suggestive of a more prominent role for the glycolytic machinery in these cells.

### Fatty Acid Metabolism in Early T helper Cell Activation

A critical aspect of early T cell activation is the upregulation of lipid metabolism, especially lipid synthesis pathways, which enables cells to grow and divide. Typically, mTORc1 promotes lipid synthesis pathways by activating SREBP transcription factors (71). One of many targets of SREBPs is an enzyme essential in *de novo* synthesis of fatty acids, ACC1. In studies by Berod et al. (72), inhibition of ACC1 genetically or pharmaceutically prevented the differentiation of all effector lineages, while Treg cells preferentially differentiated from cultures of Th17 cells. This demonstrates that fatty acid synthesis is an essential feature of early T helper cell differentiation. In a separate study, it was suggested that early T helper cell activation, proliferation and growth may also rely on fatty acid uptake, orchestrated by the nuclear receptor, peroxisome proliferator activated receptor gamma (PPAR-γ) (73). However, the early division and proliferation of T helper cells was shown to be unaffected by genetic loss of PPAR-γ in another study (74) and the increase in PPAR-γ expression under neutral conditions is minor (73, 74). PPAR-γ becomes highly expressed specifically in Th2 cells and likely regulates fatty acid metabolism later in the Th2 cell differentiation program.

## Metabolic Changes During Later Activation of Th2 Cells

### Inflammatory Cytokines in the Tissue Potentiate Th2 Cell Differentiation

Priming of CD4 T cells toward the Th2 cell subset in the lymph node induces the production of IL-4. However, several studies in the context of infection to *N. brasiliensis* and *H. polygyrus*, or to the allergen house dust mite (HDM) have shown that a large portion of IL-4-producing cells in these settings are Tfh cells (38, 75, 76). Full Th2 cell effector functions in the *N. brasiliensis* and *HDM* models, are not observed until T helper cells reach the lung tissue.

It is increasingly appreciated that the activation of epithelial and innate cells at the site of allergen, venom or pathogen entry plays an important role in shaping Th2 cell responses (77). Impaired barrier function, exposure to damage associated molecular patterns (DAMPs) and microbial products can trigger receptors like Toll Like Receptor-4 (TLR4) and Protease Activated Receptor-2 (PAR2) in epithelial and tuft cells that line the surface of the airways (78, 79). These cells as well as innate lymphoid cells (ILCs), macrophages and dendritic cells (DCs), in turn secrete a range of potent inflammatory cytokines including IL-1, IL-18, IL-25, IL-33, GM-CSF, M-CSF, and thymic stromal lymphopoietin (TSLP) (42, 77). In the case of DCs, cytokines such as TLSP and IL-33 can promote expression of OX40L and suppression of IL-12 which further promotes Th2 cell differentiation and function (80–85). These signals from epithelial cells and innate cells in the lung have been shown to contribute significantly to the final identity of Th2 cells in the tissue environment (41, 42).

Studies of infection with the helminth *N. brasiliensis* suggest that cytokines such as IL-25, IL-33, and TSLP are not required for the initial stages of Th2 cell differentiation within the lymph node but function to shape those “primed” Th2 effector cells upon entry to the inflamed sites such as the lung (42). Furthermore, our recent work depicted that activated T cells entering the lung were exposed to type I interferons in the context of HDM challenge, although how this cytokine may impact on Th2 cell functions remains unclear (41).

Comparison of the transcriptional and chromatin landscapes of lung Th2 cells to Th2 cells or naïve CD4 T cells from the lymph nodes demonstrated stark differences in lung Th2 cells (41, 42), suggesting that these cells more closely aligned with type 2 ILC (ILC2) from the lung than their lymph node counterparts (86). Thus, striking changes in cellular identity occur when T helper cells reach inflammatory tissues, and this is likely coupled with important metabolic changes.

### Activation of Lipid Metabolism Pathways Is a Prominent Feature of Tissue Th2 Cells

A distinguishing feature of Th2 cells in the airways of mice administered HDM antigens was a striking upregulation in the expression of genes related to lipid oxidation and synthesis (41) (Figure 1). Comparison of DNA accessibility by ATAC-Sequencing also revealed more open chromatin at many gene loci associated with lipid metabolism in Th2 cells, compared with other T helper cell subsets in the lung, or naïve CD4 T cells in the lymph node (41). This feature of Th2 cells appears to be shared by ILC2 in the gut and lungs (87, 88). Using etomoxir and orlistat *in vivo* to block fatty acid oxidation, synthesis and uptake, Wilhelm and colleagues demonstrated that ILC2 were highly dependent on fatty acid metabolism both for their expansion and function (88). Similarly, blockade of these pathways in Th2 cell-mediated inflammation of the airways reduced Th2 cell pathologies such as airway eosinophilia and goblet cell metaplasia, and appeared to reduce T helper cell expansion and the production of IL-5 and IL-13 by Th2 cells to some extent (41).

### PPAR-γ: Linking Th2 Cell Function and Cellular Metabolism

A feature of Th2 cells, ILC2, and alternatively activated M2 macrophages is the expression of PPAR-γ, a master regulator of adipocyte differentiation and regulator of lipid metabolism in various cell types (89–93). PPAR-γ belongs to a superfamily of nuclear receptors whose transcriptional effects are regulated by many natural ligands and dependent on co-factors such as CEBP, RXRα and other transcription factors (94). In macrophages and dendritic cells, PPAR-γ expression is induced by IL-4R ligation and STAT6 activation and it is likely the same mechanism at play in Th2 cells (95–97). The absence of PPAR-γ prevents the acquisition of the M2 phenotype, with impaired fatty acid uptake and mitochondria biogenesis (96, 98). The absence of PPAR-γ in CD4 T cells ameliorated Th2 cell-associated pathology in airway inflammation models and impaired Th2 cell-mediated immunity to *H. polygyrus* (91, 93). An important facet of this phenotype was that PPAR-γ appeared to be particularly important for the pathogenic phenotype of Th2 cells in the lung (91, 93). Early activation of Th2 cells in lung-draining lymph nodes did not appear to be greatly affected (91). This suggests that PPAR-γ becomes important in sensing ligands in inflamed tissue. It remains unclear how the loss of PPAR-γ impacts on ILC2, although its high expression specifically in this subset of ILC, and its important role in M2 macrophages and Th2 cells implies that it could be important for ILC2 functions.

### PPAR-γ Directly Promotes Th2 Cell Functions

The impact of PPAR-γ on the expression of the early Th2 cell effector cytokine IL-4 is ambiguous. Studies have characterized that the absence of PPAR-γ reduces (93), increases (74) or has no effect (91) on CD4 T cell-derived IL-4, thus making it apparent that the impact of PPAR-γ on IL-4 production is context and assay dependent. A clearer impact of PPAR-γ has been demonstrated for features of Th2 cells in lung tissue. For instance, the absence of PPAR-γ in CD4 T cells impairs the expression of ST2 in lung and airway Th2 cells, and significantly impairs the expression of IL-5 and IL-13 by CD4 T cells (91). In humans, PPAR-γ is highly expressed in CRTH2^+^ Th2 cells thought to harbor the pathogenic Th2 cell subset (93). It has also been linked to IL-9 production by a subset of pathogenic Th2 cells, which are prevalent in lesions taken from the skin of contact dermatitis patients (99). Inhibition of PPAR-γ profoundly suppressed the frequency of IL-9^+^ Th2 cell clones.

Mechanistic studies have pinpointed an enrichment for PPAR-γ binding sites at open chromatin regions in Th2 cells (41), and chromatin immunoprecipitation-sequencing (CHIP-Seq) has identified a number of critical target genes for PPAR-γ binding including *Ap1, Ets1, Runx1, Gata3, Stat5, Il5*, and *Il13* (100). Since PPAR-γ is a potent repressor as well as activator of gene transcription, it is difficult to predict the impact of this nuclear receptor through CHIP-Seq and ATAC-Seq analysis. Our own work demonstrated that the addition of PPAR-γ ligands to *in vitro* cultures had little direct impact on effector cytokine production by Th2 cells, but potently upregulated ST2 expression (91). For instance, the prostaglandin derivative 15dΔ12,14- PGJ_2_ (15d-PGJ_2_) was able to induce ST2 expression, as did synthetic agonists such as pioglitazone (101, 102), a member of the class of clinically-approved compounds known as thiazoldinediones (TZDs). Thus, PPAR-γ plays an important role in shaping the chromatin architecture of Th2 cells and appears particularly important for late stage effector functions of Th2 cells.

### A Role for PPAR-γ in Modulating Th2 Cell Metabolism

While PPAR-γ is a well-characterized regulator of cellular metabolism in macrophages, dendritic cells, tumor cells and adipocytes (103–105), its impact on Th2 cell metabolism is less well-understood. In co-operation with STAT6, PPAR-γ is thought to regulate lipid metabolism in DC and macrophages through the regulation of genes including *Fabp4* (97).

In Th2 cells, arrays of PPAR-γ-deficient ST2^+^ T helper cells suggested that a range of metabolic pathways may be affected by the absence of PPAR-γ including carbohydrate synthesis, metabolite transport, lipid storage and lipolysis (91). However, these gene expression arrays are complicated by the fact that ST2^+^ Th2 cells have difficulty differentiating into fully pathogenic Th2 cells. In the study by Angela and colleagues, PPAR-γ was shown to be induced by mTORc1 activation and particular important for the expression of genes associated with fatty acid uptake and lipolysis including *Ldlr, Scrab2, Vdlr, Plin2, and Fabp5* (73). In this study, silencing of PPAR-γ impaired oxidative metabolism and glycolysis suggesting that PPAR-γ may not only promote lipid metabolism.

Thus, PPAR-γ plays important roles in promoting the expression of critical Th2 cell-associated factors such as ST2, but also likely contributes to regulating the lipid metabolism in these cells, especially in the tissue context. More mechanistic studies are required to dissect the impact of PPAR-γ on cellular metabolism in T cells, potentially in the context of overexpression systems.

### Glycolysis and Th2 Effector Cell Function *in situ*

Glycolysis is not only important in the early phases of T cell activation but may also play a direct role in shaping T cell effector functions in inflamed tissues such as the lung. Active glycolysis has been shown to promote production of IFN-γ by Th1 cells and CD8 T cells *in vitro* and in the tumor microenvironment (106, 107). In the absence of active glycolysis, the enzyme glyceraldehyde 3-phosphate dehydrogenase (GAPDH) bound the 3'UTR of *Ifng* mRNA, impeding its translation (106). Our own recent studies depicted that following *in vivo* or *in vitro* blockade of glycolysis, the expression of IL-13 and IL-5 was significantly reduced (41). Whether this is also mediated through GAPDH remains to be determined.

High concentrations of extracellular lactate, a byproduct of glycolysis, has also been shown to reduce the CCL5-induced motility of CD4 T cells. This effect is mediated by the sodium lactate transporter Slc5a12 and has been proposed as a mechanism retaining effector cells at sites of inflammation (108). It has also been shown to potentiate CD8 T cell function (109). Whether these mechanisms play a role in Th2 cell function and the pathology of asthma remains to be shown.

In summary, while glycolysis appears to be important for effector cytokine production by T helper cells, Th2 cells in tissues appear enriched for pathways associated with lipid metabolism compared to other T helper cell subsets and naïve cells. This is typified by the expression of genes associated with fatty acid metabolism, the open chromatin state of Th2 cells at several key genes associated with lipid metabolism, and the requirement for PPAR-γ in mediating robust Th2 cell-mediated immune responses.

## The Role of Extra Cellular Metabolites on Th2 Cell Differentiation and Function

In addition to the activation of PPAR-γ by derivatives of prostaglandins and medium chain fatty acids, a range of other metabolites have been postulated to regulate Th2 cell differentiation and function.

### Extracellular ATP

ATP found in the extracellular environment operates as a potent DAMP due to its almost complete absence from healthy tissues and its quick release following cell damage (110). It is sensed via P2X and P2Y receptors, expressed throughout the immune system. It has been widely shown that levels of extracellular ATP are elevated in the bronchoalveolar lavage fluid (BALF) of asthmatic patients in comparison to healthy controls (111) and it is thought to induce migration of eosinophils and activation of mast cells in the lung and airways (112). Unlike Treg cells, Th2 cells appear to be relatively insensitive to cell death induced by extracellular ATP (113). Exposure of mice to inhaled allergens such as ovalbumin (OVA) results in an increase of ATP in the airways (111). Non-degradable forms of ATP, which cannot be metabolized by ectonucleases CD39 and CD73, are capable of breaking tolerance and inducing type 2 responses to inert antigens such as OVA (111). It can also be induced in airway epithelium in response to allergens such as the Cockroach allergen, Per a 10, or aeroallergens derived from *Alternaria alternata*. It can drive IL-33 release which further supports Th2 cell differentiation and metabolic reprogramming *in situ* (114, 115). Autocrine ATP is sensed via the P2Y_2_ receptor, which increases intracellular Ca^2+^ concentrations that in turn increase IL-33 release. Blockade of the P2Y_2_ receptor is sufficient to halt Th2 cell induction (115). ATP has also been shown to induce DCs that promote Th2 cell responses. Interestingly, CD39-deficient mice have defective Th2 cell responses to both OVA and HDM (116). In the absence of CD39, DCs in these mice have impaired purinergic receptor activity, appear less able to upregulate co-stimulatory molecules and exhibit defects in chemotaxis. These studies suggest an important inflammatory role for ATP in driving Th2 cell responses to a range of allergens.

### Short Chain Fatty Acids (SCFAs)

The gut microbiome greatly influences the composition of metabolites that is in our circulation. One important immune regulatory product of fermenting bacteria in the intestines are SCFAs. SCFAs can be transported into cells via various receptors and have been shown to contribute to epigenetic modifications (117). SCFAs also bind to G-Protein Coupled Receptor 41 (GPR41, also known as FFAR3) and GPR43 (FFAR2) (118) and can modulate immune cell functions through these receptors. Typically, SCFAs have been shown to suppress inflammation and promote tolerance by various mechanisms. In line with this, mice fed a high fiber diet and hence with high circulating levels of SCFAs, have been shown to develop reduced airway inflammation in OVA/alum and HDM models of allergic airway disease. In one study, SCFAs appeared to suppress dendritic cell activation and migration through GPR41 (118). In another study, GPR41 was proposed to promote Treg cell IL-10 production, thereby suppressing allergic airway inflammation (119). Administration of a high-fiber or high-acetate diet in pregnant mice was also shown to reduce allergic airway disease in progeny, by promoting acetylation of the Foxp3 locus in Treg cells, highlighting the potent immune modulatory effects of these molecules (120). Studies of ILC2 have also shown that type 2 cytokine production and GATA3 expression may be dampened by SCFAs (121, 122). Despite the known anti-inflammatory function of SCFAs, a recent study of Th2 cells in people with eosinophilic esophagitis and in a mouse model of fungal infection revealed that SCFAs may also potentiate Th2 cell cytokine production (123). Thus, the impact of SCFAs on Th2 cell differentiation and function requires further investigation.

### Glutamine

The conditionally essential amino acid glutamine is found at relatively high concentrations in the plasma and is capable of providing cells with a potential source of energy. However, under conditions of catabolic stress such as tissue damage or infection demand for glutamine rises with immune cells consuming particular high levels (124). Upon activation, T cells increase their uptake of glutamine 5–10-fold through the glutamine specific transporters SNAT-1/2 (124, 125). It serves as an important source of nitrogen and as an anapleurotic substrate for the TCA cycle and production of ribose in T cells (126–128). A lack of glutamine results in a failure for sustained proliferation and impairs cytokine release from T cells (125). Although glutamine plays an essential role in T cell activation, the addition of glutamine to the diet has been shown to favor Th1 cell responses over Th2 cells (129). Similarly, addition of high concentrations *in vitro* impairs Th2 cell differentiation in human PBMC cultures (130). This is at least in part due to the ability of high concentrations of glutamine to inhibit cytosolic phospholipase A2 (cPLA2), a key enzyme in releasing arachidonic acid from glycerophospholipids (131). This in turn provides the precursor molecules for a number of eicosanoids including well-known inflammatory mediators such as leukotrienes (LTs), prostaglandins and platelet-activating factor (PAF), which are important for Th2 cell functions. Thus, glutamine has the ability to regulate Th2 cell responses.

### Indoamine 2,3-dioxygenase (IDO)

IDO is the rate-limiting enzyme required for tryptophan (TRP) metabolism (132). Within tissues such as the lung, IDO expression is high on epithelial cells and certain DC subpopulations (133). Given its constitutively high expression on Treg cells and cancer, IDO has been widely linked to immune suppression although some studies have indicated that it can in fact promote Th2 cell function. For instance it has been observed that 3-hyrdroxyanthranilic and quinolinic acids, metabolites of the KYN pathway, are capable of inducing apoptosis in Th1 cells without affecting Th2 cells (134). IDO expression by eosinophils is capable of inhibiting IFN-γ production by Th1 cells with no effect on Th2 cell function (135).

It has also been shown that IDO can potentiate Th2 cell cytokine production during *in vitro* differentiation of Th2 cells (133). In the context of airway inflammation, IDO-deficient mice appeared to have reduced Th2 cell responses and reduced levels of circulating IgE. Thus, IDO may aid in potentiating the polarization of Th2 cells and possibly inhibit bystander Th1 cells. Reduced expression of IDO during pregnancy results in enhanced ratios of Th1:Th2 cells (136). Interestingly, KYN-TRP levels are profoundly influenced by the composition of the gut microbiota via activation of AhR and TLRs in the host (137). Thus, regulation of TRP metabolism by IDO appears to promote Th2 cell responses.

## Metabolic Interventions Targeting Type 2 Inflammation

Clinical and epidemiological studies have long indicated that type 2 inflammation, allergies and metabolic disorders are highly linked. For instance, a strong link between obesity and asthma has been reported in many studies (138, 139). In addition to BMI, other abnormalities in metabolism are also thought to predispose children and adults to asthma (138, 139). One obvious solution to reducing asthma linked to obesity is weight loss through exercise, although it can be difficult for asthmatics due to exercise-induced exacerbations. Nonetheless, aerobic exercise is known to reduce lung and airway inflammation, and reduce pathogenic cytokine secretion (140), which suggests that the right form of exercise could alleviate symptoms of asthma, in some patients.

Despite recent evidence that PPAR-γ promotes Th2 cell functions in mice and humans (91, 93), preclinical studies had repeatedly shown that PPAR-γ agonists reduced goblet cell metaplasia, alarmin release and airway hyperresonsiveness in mouse models of asthma (141–144). For this reason, several trials of TZDs were initiated in asthma and COPD. However, one recent trial using pioglitazone resulted in exacerbations in 14% of severe asthmatics. No patients experienced improvements in their disease symptoms, resulting in a premature cessation of this trial (145, 146).

In the last few years, a number of trials of putative anti-inflammatory dietary compounds has been initiated. A recent trial of polyunsaturated fatty acids conducted in pregnant women in Denmark showed that infants born to mothers on this supplement had a reduced absolute risk of developing wheeze and asthma in the first 3 years of life (147). Thus, modulating inflammation through the diet of mothers shows promise as a way to prevent allergy in infants. Furthermore, the anti-inflammatory effects of high fiber diets in preclinical studies have led to the commencement of trials in various disease settings including diabetes and asthma (148, 149). The results of these trials are eagerly anticipated.

## Concluding Remarks

As Th2 cells differentiate, their metabolic requirements and exposure to nutrients changes dramatically. In the early activation of CD4 T cells, strong induction of glycolysis and lipid metabolism is required to kick start the differentiation of effector T helper cell lineages including Th2 cells, seemingly at the expense of Treg cell differentiation. These pathways drive cell division and proliferation in essentially all T helper cell subsets, although mTORc2 appears to promote Th2 cell differentiation via several specific mechanisms. The extracellular environment changes profoundly when Th2 cells move from the draining lymph node to inflamed tissues via the vasculature. In the blood and in non-lymphoid tissues, T helper cells become exposed to levels of glucose, lipids, SCFAs and amino acids that are known to vary highly between individuals, and can have an important impact on Th2 cell differentiation. Important sensors for these factors include solute carrier proteins, ectonucleotidases, G-protein coupled receptors and nuclear receptors like PPAR-γ. In tissues, Th2 cells appear insensitive to death induced by ATP-sensing receptors, rely on lipids either as a source of energy or as ligands for PPAR-γ, and are susceptible to regulation by SCFAs released by the microbiota. Hence, sensors of the extracellular environment influence the metabolism and function of T helper cell subsets in peripheral tissues and can have a strong bearing on the cytokine balance of an individual.

A major challenge going forward is whether we can understand precisely how all of the metabolic components in our blood and tissues work together to regulate T helper cell responses in humans. An issue with current studies in metabolomics is that they are conducted in patients with disease, who can have metabolic disruptions for multiple reasons. An important goal for the future is to conduct prospective cohort studies of healthy individuals, in order to understand how the metabolome shapes the T helper cell balance and impacts on the development of allergic diseases.

## Author Contributions

All authors listed have made a substantial, direct and intellectual contribution to the work, and approved it for publication.

### Conflict of Interest

The authors declare that the research was conducted in the absence of any commercial or financial relationships that could be construed as a potential conflict of interest.
